# Targeting mutations in cancer

**DOI:** 10.1172/JCI154943

**Published:** 2022-04-15

**Authors:** Michael R. Waarts, Aaron J. Stonestrom, Young C. Park, Ross L. Levine

**Affiliations:** 1 Gerstner Sloan Kettering Graduate Program in Biomedical Sciences,; 2 Human Oncology and Pathogenesis Program,; 3 Center for Hematologic Malignancies,; 4 Center for Epigenetics Research, and; 5 Leukemia Service, Department of Medicine, Memorial Sloan Kettering Cancer Center, New York, New York, USA.

## Abstract

Targeted therapies have come to play an increasingly important role in cancer therapy over the past two decades. This success has been made possible in large part by technological advances in sequencing, which have greatly advanced our understanding of the mutational landscape of human cancer and the genetic drivers present in individual tumors. We are rapidly discovering a growing number of mutations that occur in targetable pathways, and thus tumor genetic testing has become an important component in the choice of appropriate therapies. Targeted therapy has dramatically transformed treatment outcomes and disease prognosis in some settings, whereas in other oncologic contexts, targeted approaches have yet to demonstrate considerable clinical efficacy. In this Review, we summarize the current knowledge of targetable mutations that occur in a range of cancers, including hematologic malignancies and solid tumors such as non–small cell lung cancer and breast cancer. We outline seminal examples of druggable mutations and targeting modalities and address the clinical and research challenges that must be overcome to maximize therapeutic benefit.

## Introduction

Advances in the classical cancer treatment modalities of standard cytotoxic chemotherapy, radiotherapy, and surgery have led to reductions in cancer mortality rates over the last several decades; however, major challenges remain that often lead to tumor recurrence and death. These challenges have led to the exploration of mutation-targeted therapies for cancer. While standard chemotherapy uses cytotoxic agents that kill rapidly dividing malignant and normal cells, targeted therapies act on abnormal proteins encoded by mutated genes. Since normal cells lack the tumorigenic mutations that are exploited for drug targeting, there is often high differential sensitivity of malignant and nonmalignant cells to targeted therapies. As a result, targeted therapy frequently produces rapid and dramatic tumor regression while limiting the potential for off-target toxicities that are a hallmark of conventional chemotherapy. The overall drug discovery strategy for cancer has thus shifted away from cytotoxic agents and toward the identification of tumor-specific actionable mutations and the development of molecularly targeted agents. The rapid development of immune-based therapies has also dramatically changed the cancer therapeutic landscape but is outside the scope of this Review (1).

Progress in targeted therapies is closely tied to technological advancements in sequencing made over the past two decades, including the revolutionary development of massively paralleled next-generation sequencing (NGS) (2). The discovery of both common and rare genetic aberrations has launched research investigations into targeted therapies against resultant mutant proteins and exploration of downstream aberrant molecular signaling pathways that can be therapeutically exploited (3). Additionally, NGS has proved critical to the clinical application of targeted therapies. Mutational evaluation by sequential oligonucleotide capture, amplification, and NGS has become a standard-of-care diagnostic tool in many cancers (4, 5). These tests are used to identify actionable genetic mutations that are then used for selection of an appropriate targeted therapy, for prognostication, or as biomarkers for other clinical endpoints.

**Figure 1:**
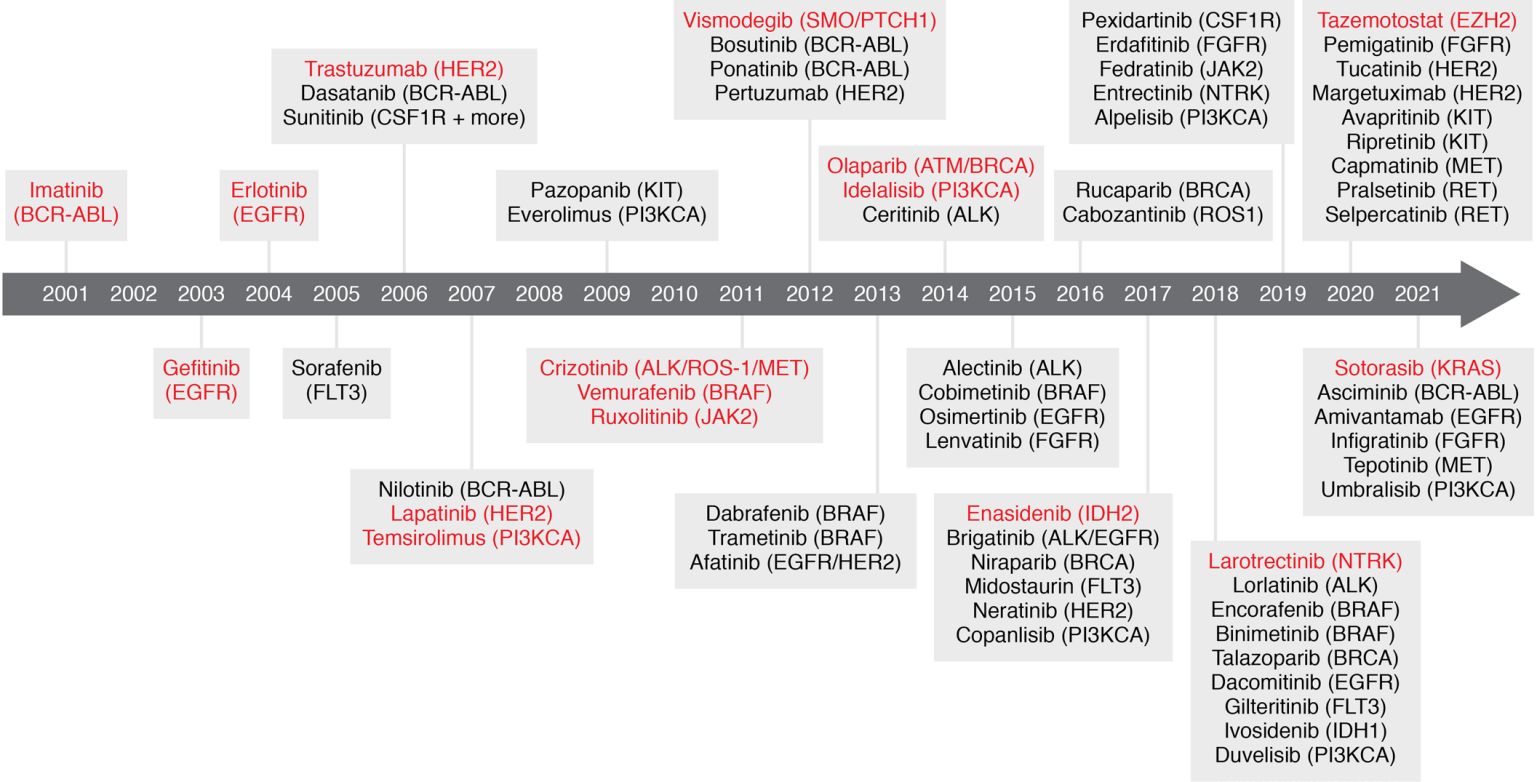
Timeline of FDA-approved targeted therapies in cancer. FDA-approved molecules with mutation-specific indications are depicted along with their associated mutation-specific indications. First-in-class molecules for novel targets are shown in red.

The discovery of the *BCR-ABL* fusion gene as the hallmark of chronic myelogenous leukemia (CML) and the development of the BCR-ABL inhibitor imatinib marked a pivotal moment in drug development. Since then, numerous targeted therapies have been approved by the US FDA, with many more agents under investigation (Figure 1). Oncogenic gene mutations may be druggable in several different ways: (a) they can encode a protein that can be targeted in a manner distinct from the WT protein; (b) they can cause abnormal activation of a protein (e.g., through a gain-of-function mutation or amplification) that is druggable but for which mutant-specific targeting has not been achieved; or (c) they can create novel molecular dependencies that are druggable (“actionable mutations”). Examples of truly druggable mutations in the first category are far less common than druggable mutations in the second category. However, excellent therapeutic indices are still achieved for many overactivated or amplified targets due to increased target expression and/or a high level of cancer-specific dependence on a specific protein. Additionally, other mutations that are not yet amenable to targeted therapy approaches are still useful as biomarkers that support selection of another appropriate therapy. Biomarkers are reviewed extensively elsewhere; here, we focus on progress made on mutations directly linked to targeted therapies (6, 7). We describe key examples of genetic drivers and associated modes of druggability, consider the clinical and research challenges in the field, and discuss new approaches to maximizing therapeutic benefit of targeted therapies. 

## Key mutations with actionable therapies

Targetable genetic aberrations occur in a range of genes encoding kinases and their downstream signaling effectors, tumor suppressors, and chromatin modifiers. Table 1 lists common actionable genetic aberrations associated with FDA-approved targeted therapies. Below, we highlight representative examples of genetic aberrations and corresponding targeted therapies that demonstrate the benefits targeted therapy can provide and the challenges that must be overcome in designing novel therapies. 

### Tyrosine kinases.

**Figure 2:**
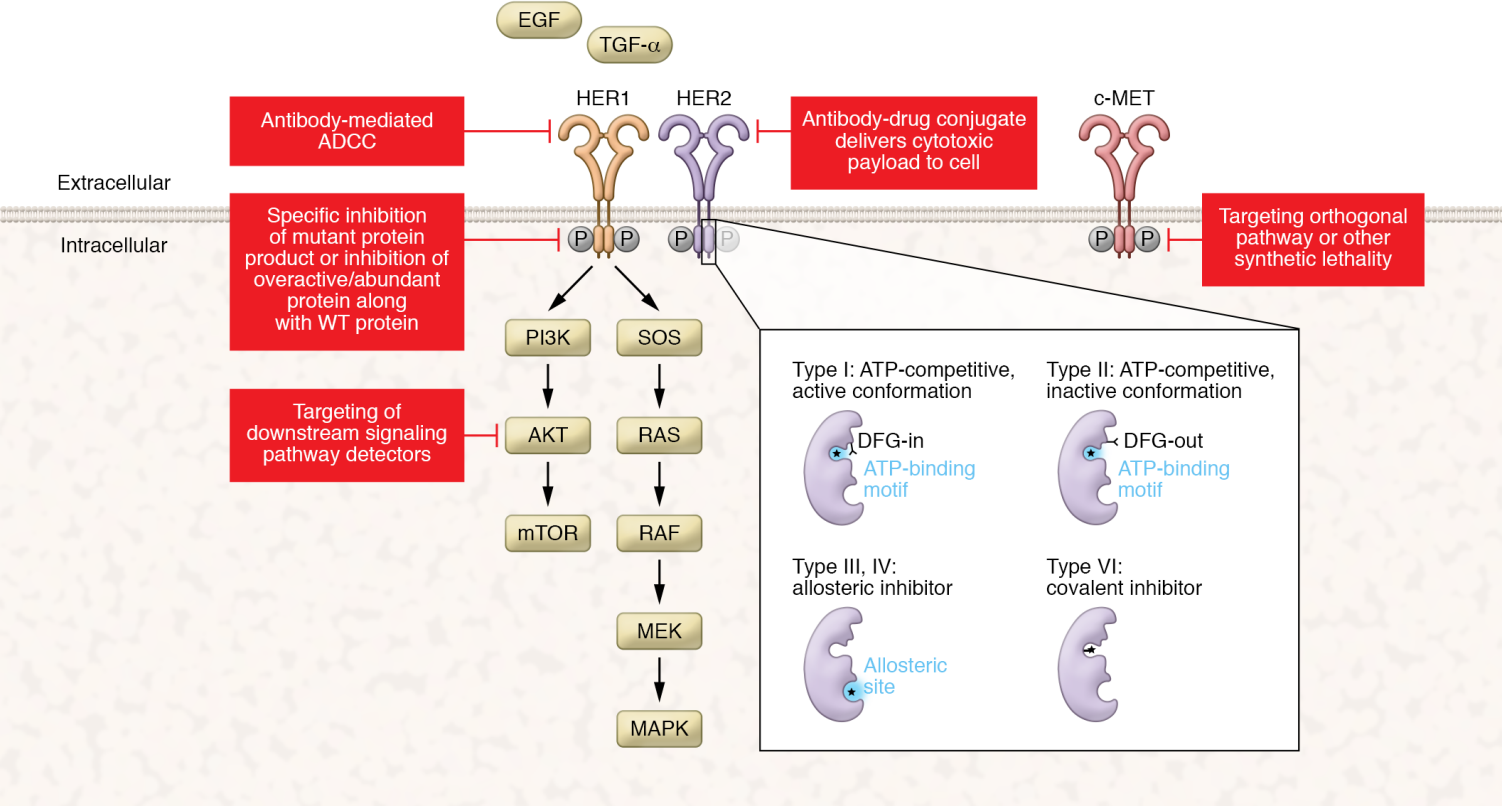
Targeting modalities for targeted therapies in cancer. Common modes of druggability associated with clinically approved molecules are depicted using the HER1/HER2 RTKs as examples. FDA-approved targeted therapies include small-molecule inhibitors and mAbs. Small-molecule inhibitors are commonly classified on the basis of the mechanism by which they bind their targets (inset). Small-molecule inhibitors in cancer can directly inhibit a mutant protein product, inhibit an overactive/overabundant protein product along with WT protein, or inhibit a signaling effector downstream of a mutated protein. In addition to inhibitors, mAbs are approved either with or without the addition of a drug conjugate, which, besides activating antibody-dependent cellular cytotoxicity (ADCC), also delivers cytotoxic payloads to targeted cells. DFG, aspartate-phenylalanine-glycine motif.

Following the success of imatinib, there has been an enormous amount of interest in targeting other mutated kinases. As developments in sequencing have added to the number of candidate targets, parallel progress in chemical approaches has expanded the number of targets considered druggable. Kinases can be divided into two general types: tyrosine kinases, which act primarily as growth factor receptors or in direct interaction with growth factor receptors, and serine/threonine kinases, which respond to a range of cellular cues, including signaling downstream of tyrosine kinases. Kinase inhibitors may act through any of multiple mechanisms, including by competition for ATP in the ATP-binding pocket of the kinase in either active (type I) or inactive (type II) conformation, allosteric inhibition of kinase activity (types III and IV) through binding of other regions of the kinase, and/or other mechanisms (Figure 2 and refs. 8, 9). For tyrosine kinases, in addition to chemical inhibitors, monoclonal antibodies (mAbs) targeting the extracellular domains have emerged as a promising therapeutic approach (10, 11). Here, we begin by discussing several examples of mutations in tyrosine kinases that have been successfully targeted with different strategies. 

In 2001, the tyrosine kinase inhibitor (TKI) imatinib became the first FDA-approved targeted therapy for a known genetic alteration. Imatinib’s initial approval was for CML, characterized by the presence of the Philadelphia chromosome, a molecular juxtaposition of the tyrosine kinase *ABL1* on chromosome 9 to *BCR* on chromosome 22 (12). The resultant fusion gene encodes an oncoprotein that is capable of autophosphorylation and constitutive activation of downstream signaling pathways including PI3K/AKT/mTOR, MAPK, and JAK/STAT, leading to unrestrained cell proliferation (13, 14). Before the development of imatinib, the 10-year survival rate of the approximately 90% of patients presenting with chronic-phase CML was less than 50%. Extraordinarily, treatment with imatinib increased the 10-year survival rate to approximately 80% (15, 16). Despite its success, two challenges with imatinib are resistance and side effects. Resistance occurs through several mechanisms, most commonly due to mutations in the kinase domain of BCR-ABL (17–19). These mutations may develop while patients are on imatinib but may also be present in the CML prior to the start of treatment and may be selected for by insufficient drug levels (20). To overcome resistance, next-generation kinase inhibitors with increased inhibitory activity toward BCR-ABL have been developed. Nilotinib, dasatinib, bosutinib, and ponatinib compete in the same ATP-binding pocket as imatinib but with higher affinity and are able to overcome many resistance mutations (21–24). More recently, the allosteric inhibitor asciminib has also been approved (25). Because asciminib does not bind in the ATP-binding pocket of BCR-ABL but instead binds in the myristylation pocket that is responsible for maintaining the kinase in the autoinhibited state, asciminib is expected to overcome nearly all known resistance mutations (26). Additionally, because the binding site of asciminib is entirely distinct from that of other BCR-ABL–targeted TKIs, combination targeting of BCR-ABL activity is possible and may prevent the acquisition of resistance (27). Despite excellent efficacy, TKI therapy is generally given lifelong, as it appears that CML stem cells can persist at low levels in the presence of therapy, possibly due to quiescence or activation of other signaling pathways (28). Side effects of CML therapy are common and have particularly detrimental effects on quality of life given lifelong treatment.

**Figure 3:**
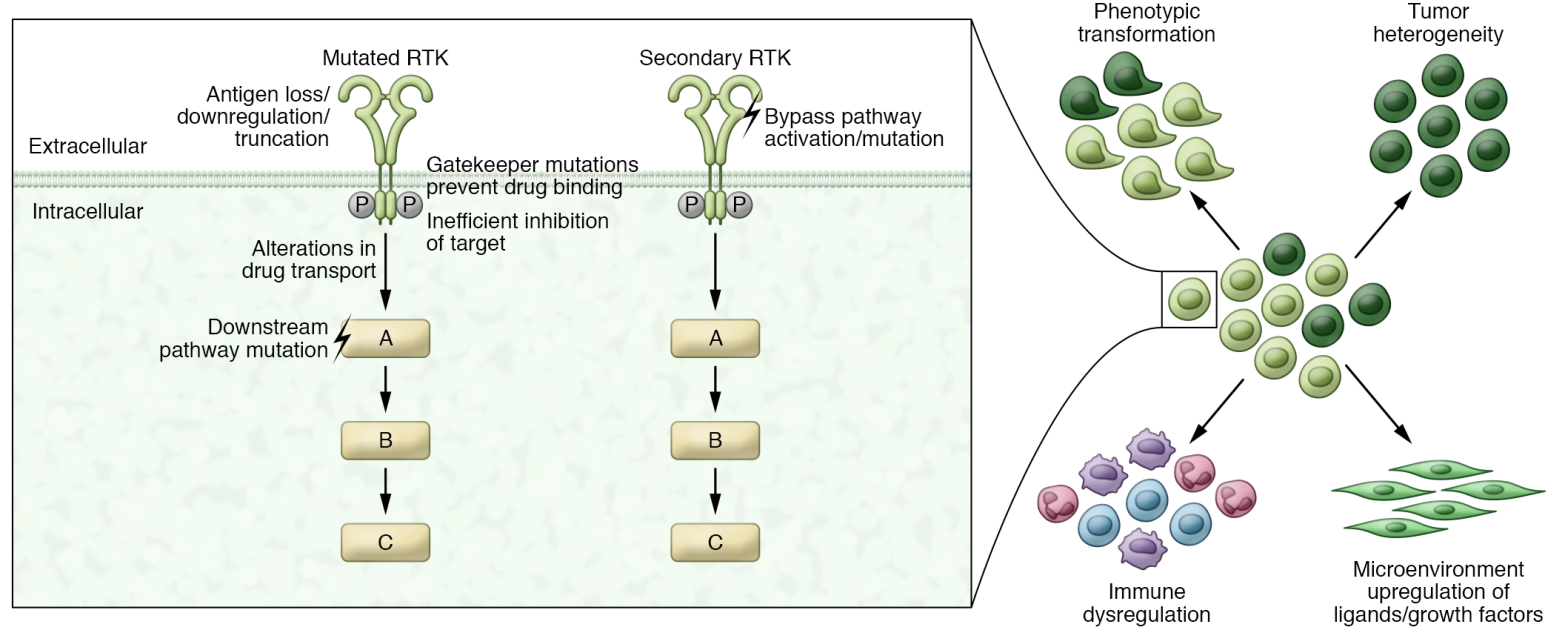
Mechanisms of resistance to targeted therapies against kinases in cancer. In response to kinase inhibitors, mutations in the kinase domain that prevent drug binding to the target are the most frequent resistant mechanisms. Other mechanisms of resistance to kinase inhibition include alterations in drug transport or metabolism and mutations in either downstream pathway effectors or alternative signaling pathway effectors. Resistance also occurs with mAbs targeting kinases, with loss/downregulation/truncation of the targeted antigen being the most common. Resistance mechanisms that affect both small-molecule inhibitors and mAbs are also common and include phenotypic transformation, tumor heterogeneity, immune dysregulation, and microenvironmental upregulation of ligands/growth factors.

Drugs targeting the EGF/human EGFR (HER) family of receptor tyrosine kinases (RTKs) were the second large group of targeted therapies to be approved by the FDA. Under normal physiologic conditions, ligand binding to the extracellular domains of HER RTKs causes dimerization of receptors leading to autophosphorylation within the intracellular tyrosine kinase domains and consequent activation of downstream effectors (29). Mutations of HER family members result in constitutive downstream pathway signaling and are found across a spectrum of cancers; the most common mutations include *EGFR*/*HER1* activating mutations in non–small cell lung cancer (NSCLC) and *HER2* amplifications/activating mutations in breast cancer (BC) (3). Both EGFR and HER2 TKIs have been developed; TKIs against the latter are less widely used but have clinical efficacy in specific settings and are reviewed elsewhere (30). Early-generation EGFR inhibitors block both mutant and WT EGFR signaling reversibly (gefitinib/erlotinib) or irreversibly (afatinib/dacomitinib) (31–34). Patients treated with these drugs often have dramatic initial responses to therapy but develop resistance mutations including T790M and experience side effects due to inhibition of WT EGFR. The development of the covalent EGFR inhibitor osimertinib represented a major step forward due to increased relative affinity for mutant EGFR over WT and retention of efficacy against the T790M mutation (35, 36). Nevertheless, resistance eventually occurs in all patients with stage IV *EGFR*-mutant NSCLC. While a subset of resistance occurs through mutations at osimertinib’s reactive cysteine target C797, it is also common for resistance to occur through mechanisms that do not depend on specific mutations, such as histologic small cell transformation (Figure 3 and refs. 37, 38).

Targeting HER family members with mAbs is another attractive therapeutic option. mAbs bind to the juxtamembrane portion of the extracellular domain of RTKs and act through multiple mechanisms, including antibody-dependent cellular cytotoxicity (ADCC), RTK internalization/degradation, and inhibition of RTK dimerization (10, 11). The first clinically approved HER2-targeting mAb, trastuzumab, has exceptional activity in HER2-positive BC (39). Pertuzumab is another HER2 mAb that targets a different site on HER2 and is able to disrupt ligand-induced dimerization of HER2, thus also inhibiting its receptor partner HER3 (40). Since the binding sites of trastuzumab and pertuzumab are distinct, these agents are often combined to maximize efficacy (41). A third HER2-targeting mAb, margetuximab, has improved ability to recruit effector immune cells and may retain efficacy after resistance to other mAbs has developed (42). HER2-targeted antibody-drug conjugates that deliver cytotoxic payloads have also been developed, including trastuzumab-emtansine, trastuzumab-deruxtecan, and trastuzumab-duocarmazine (43). EGFR mAbs, including cetuximab, panitumumab, and necitumumab, have shown limited activity in NSCLC but have specific indications in the treatment of colorectal cancer and squamous cell carcinoma of the head and neck (44). Finally, bivalent mAbs targeting two oncogenic drivers are a further therapeutic strategy, and recently the bivalent mAb amivantamab targeting EGFR and MET has been approved for NSCLC (45). While mAbs have proved clinically effective, many patients ultimately develop resistance to these agents due to immune-mediated escape from ADCC, intrinsic alterations in the RTK extracellular domain, and activation of alternative signaling pathways (46–49).

Targeting of mutations in *FGFR*/*PDGFR*/*VEGFR* family members is another area that has generated considerable clinical interest and has led to FDA approval of over a dozen drugs. Genetic aberrations in *FGFR1/2/3* occur in more than 10% of breast, bladder, and endometrial cancers as well as at lower frequencies in other tumors (3). Mechanistically, activation of the FGFR/PDGFR/VEGFR pathways occurs through a process similar to that of activation of the HER RTKs, and mutations in these pathways likewise act to cause constitutive activation of the mutated RTK (50). The primary and most well-studied downstream effect of FGFR/PDGFR/VEGFR signaling is increased angiogenesis. Most inhibitors against this class of RTKs to date have activity against all three RTK families and are more frequently used in a mutation-agnostic practice to limit angiogenesis rather than being employed for mutation-specific cancers (51). While effective in certain contexts, these multikinase inhibitors have overall achieved modest clinical results, for multiple reasons including insufficient target inhibition and increased toxicity owing to multikinase inhibition. FGFR-specific inhibitors, including erdafitinib, infigratinib, and pemigatinib, have been developed more recently but have also generated limited responses (52–54). The presence of an *FGFR* alteration alone is likely not sufficient for patient selection, since only a minority of patients with *FGFR* alterations respond to TKIs and a subset of *FGFR*-WT patients also respond. Preliminary data from clinical trials indicate that FGFR expression levels may be a superior predictor of response, and the development of further biomarkers will likely be necessary to guide patient selection (55). *FGFR*-mutant tumors may also have other as-yet unidentified alterations that prevent addiction to FGFR, and it is also possible that even the existing specific FGFR-targeting therapies may not be sufficiently potent and specific to elicit mutant-specific responses (56). 

Overall, targeting mutations in tyrosine kinases has been a fruitful therapeutic strategy: BCR-ABL inhibitors have transformed treatment of CML, and both mAbs and TKIs are standard-of-care for *EGFR*-mutant NSCLC and HER2-positive BC. A variety of other TKIs (Table 1) also now play important roles in the treatment of cancers with *JAK2* and *FLT3* mutations in hematologic malignancies and *ALK*, *MET*, *NTRK*, *RET*, and *ROS1* mutations in multiple solid tumors. Both primary and acquired resistance remains a challenge in targeted therapy, with most patients experiencing disease progression. Common themes of resistance are generally at play with TKIs and include mutations within the tyrosine kinase domain of the targeted protein that prevent drug binding, mutations in downstream signaling effectors, activation of alternative signaling pathways, and histologic transformation (Figure 3). A further challenge has been to dissect the mechanisms of multi-targeted kinase inhibitors in particular tumors. Kinase inhibitors vary in their specificity and potency, with early inhibitors being mostly multi-targeted and more recent inhibitors having increased specificity and potency against single targets; however, no kinase inhibitor is completely specific, and the physiologic action is multikinase inhibition at least to some extent. Multikinase inhibitors have shown efficacy in certain contexts, and the lack of specificity has indeed facilitated their clinical use against multiple targets, but it has often been difficult to understand the specific target(s) responsible for therapeutic benefit, making the improvement of these inhibitors challenging. Considerable research is necessary to understand the precise mechanisms of TKI response and resistance and to guide the development of the next generation of inhibitors.

### Downstream signaling effectors.

Tyrosine kinases relay signaling from extracellular ligands to downstream signaling effectors. These effectors predominantly include serine/threonine kinases but also include other important proteins such as RAS. As highlighted in examples above, mutations in numerous tyrosine kinases are able to hijack downstream signaling pathways to drive oncogenesis. In addition, mutations in downstream signaling effectors themselves are also common in cancer. For mutations in serine/threonine kinases, similar therapeutic strategies to those used to target tyrosine kinases have been developed, with the exception that mAb approaches are only effective against kinases with extracellular domains. For mutations in non-kinase effectors, such as RAS, other targeting strategies have been explored. In addition to specifically targeting cancers defined by mutations in these effectors, inhibitors against these central pathways have also been used across a variety of genetically unselected tumors, with varying success.

Members of the MAPK signaling pathway are frequently mutated in cancer and include the *RAS* GTPases and the *RAF*/*MEK*/*ERK* serine/threonine kinases (57). RAS is a plasma membrane–localized GTPase that cycles between its GDP-bound inactive state and its GTP-bound active state. In response to ligand binding, RTKs undergo autophosphorylation of sites that serve to recruit and activate RAS. RAS then initiates a signaling cascade whereby RAF, MEK1/2, and ERK1/2 are in turn phosphorylated and activated. ERK1/2 then translocates to the nucleus to phosphorylate a range of substrates, leading to changes in gene expression and cellular functions. Gain-of-function mutations in *A/B/C-RAF*, *MEK1/2*, and *RAS* are all common in cancer (3). Of the serine/threonine kinases, mutations in *BRAF* are the most common and in the majority of cases affect the V600 residue, producing a mutant BRAF protein that is able to constitutively signal as a monomer independently of RAS (58). Sorafenib, a multikinase inhibitor, was the first RAF inhibitor investigated but had limited clinical activity, likely owing to a weak affinity for BRAF at clinically achievable concentrations (59, 60). The more recent RAF inhibitors vemurafenib, dabrafenib, and encorafenib have higher potency and are able to inhibit signaling by the monomeric V600E-mutant BRAF protein (61–63). However, RAF inhibitors cause cutaneous lesions in many patients as a result of paradoxical increases in RAF activity and MAPK signaling in normal skin (64–66). Addition of MEK inhibitors to RAF inhibitor therapy simultaneously abrogates this side effect while increasing MAPK suppression and prolonging responses in melanoma (67–69). However, in some tumors, even with combined RAF/MEK inhibition, resistance occurs with sustained dependence on MAPK signaling, indicating that combination therapy may still be ineffective at achieving full inhibition (70). In addition to mutations in *A/B/C-RAF*, mutations in *MEK1/2* also occur but are not sensitive to current MEK inhibitors, which are allosteric inhibitors that bind to a pocket adjacent to the catalytic site of MEK (71). 

The *RAS* family genes *NRAS*, *KRAS*, and *HRAS* are among the most commonly mutated oncogenes in many different cancers (3). Until recently, RAS itself has largely been viewed as “undruggable” since RAS lacks a hydrophobic druggable pocket and binds its ligand GTP at extremely high affinity (72, 73). Historically, efforts to target *RAS* mutations have focused on preventing RAS localization to the plasma membrane. In order to be targeted to the plasma membrane, RAS must undergo several modifications, including prenylation by FTase or GGTase. FTase inhibitors have been tested extensively in clinical trials but have largely failed in *KRAS*/*NRAS*-mutant tumors because of compensatory modification by GGTases (74, 75). However, for *HRAS* mutations, the FTase inhibitor tipifarnib has shown promise and has been granted FDA breakthrough designation (76). Recently, the attention has shifted toward directly targeting RAS-mutant proteins. Mutations in *RAS* largely occur at the positions G12, G13, and Q61, and the resultant mutant proteins vary in their rates of intrinsic GTP hydrolysis and nucleotide exchange. Of these mutants, the *KRAS^G12C^* mutation has proved amenable to targeting because of its relatively normal rate of GTP hydrolysis and the presence of a reactive cysteine residue (77, 78). Allosteric inhibitors, including sotorasib and adagrasib, have been developed that bind in a pocket specific to the GDP-inactive state, stabilizing it and preventing activation of RAS (79–81). While effective, the durability of KRAS G12C inhibitors is generally short-lived. Resistance occurs through activation of RTKs and mutations in other *RAS* isoforms (82). Targeting of other *RAS* mutations, including those in *HRAS* and *NRAS*, has thus far remained elusive owing to their high rates of GTP hydrolysis and lack of cysteine residues, although several recent studies have suggested novel targeting strategies (83).

The PI3K/AKT/mTOR pathway is another central signaling pathway in cancer whose effectors are frequently mutated. Under normal physiology, ligand binding to RTKs leads to PI3K targeting to the cytoplasmic domains of RTKs and consequent activation of the catalytic subunit of PI3K (84). PI3K then phosphorylates the plasma membrane lipid substrate PIP2 to form PIP3, which recruits AKT, facilitating its activation. AKT then phosphorylates and activates a host of downstream regulators, including the metabolic receptor mTOR. The PI3K/AKT/mTOR pathway is negatively regulated by PTEN, a phosphatase that dephosphorylates PIP3. Activating mutations and amplifications in *PI3K*, *AKT*, and *mTOR* are common, along with inactivating mutations in *PTEN* (3). Inhibitors of PI3K have been most widely used, and FDA-approved drugs include idelalisib, copanlisib, duvelisib, umbralisib, and alpelisib (85–90). Of these, only alpelisib is used for mutation-specific targeting (*PI3KCA*-mutant BC). Alpelisib was tested in a phase III clinical trial for advanced BC in patient populations with and without *PI3KCA* mutations and was found to be effective only in patients with a *PI3KCA* mutation (89). However, other PI3K inhibitors have mutation-agnostic anticancer activity (85, 86). AKT inhibitors have not yet been approved by the FDA, but capivasertib has shown promise in phase II clinical trials, a benefit dependent on the presence of a mutation in *PI3K*/*AKT*/*mTOR* (91). mTOR inhibitors, including everolimus and temsirolimus, have been FDA approved for a variety of indications, but none selective for a genetically defined patient population (92). Despite several clinical trials, response to mTOR inhibition has not been shown to correlate with the presence of any specific pathway mutations (93, 94).

Many serine/threonine kinase inhibitors have shown limited clinical efficacy, and the stratification of patients based on tumor genetics has proved complicated. Why subsets of patients with or without specific pathway mutations either respond or do not respond to the indicated targeted therapies remains unclear, and more research will be necessary to elucidate this. Nevertheless, success stories exist, such as with RAF and MEK inhibitors for BRAF V600–mutant tumors and the recent development of KRAS G12C inhibitors. Moreover, the range of mutant effector proteins that have been drugged is expanding, and in addition to inhibitors of BRAF V600E and KRAS G12C, advances in chemistry have spurred the development of small molecules targeting other *RAF* and *RAS* mutations. As with TKIs, resistance similarly occurs through multiple mechanisms, many of which are poorly understood. Combination therapy, as successfully illustrated by dual BRAF/MEK inhibition, by targeting of two or more effectors in either the same or an orthogonal signaling pathway will likely be an important strategy in treating and preventing resistance. The combination of targeted therapies with standard chemotherapy has also been explored (e.g., trastuzumab and chemotherapy) and may be beneficial in certain contexts, although careful therapy selection will be required to avoid toxicities.

### Tumor suppressors.

Tumor suppressors are among the most common genes mutated in cancer, but targeting them is particularly challenging, because functional restoration of a mutant protein product is generally more difficult than its inhibition. Nevertheless, some clinical success has been achieved. To date, FDA-approved targeted therapies for mutations in tumor suppressors have been designed to exploit synthetic lethality conferred by these mutations. Other therapeutic strategies are also being explored, although most remain in early-stage clinical trials. Here, we discuss representative examples illustrating the successes and challenges with targeted therapies against tumor suppressors. 

Mutations in *BRCA1/2* are common, predominantly in breast and ovarian cancers, and provide an example of success in developing a molecularly targeted therapy for a mutation in a tumor suppressor. BRCA1 and BRCA2 are involved in maintaining genome integrity by the repair of double-strand breaks (DSBs) through homologous recombination (HR) (95). Mutations in *BRCA1/2* have been shown to confer sensitivity to PARP inhibitors (96). PARP1 is a DNA damage sensor that binds to single-stranded DNA breaks and synthesizes poly(ADP-ribose) (PAR) chains on itself and target proteins in the vicinity of the DNA breaks, resulting in the recruitment of additional DNA repair effectors. PARP inhibitors compete with the PARP1 cofactor NAD^+^, inhibiting PARP catalytic activity, “trapping” PARP on damaged DNA, and resulting in replication fork stalling and DSBs. In an HR-competent cell, HR proteins including BRCA1/2 are then recruited for DNA repair. However, *BRCA1/2*-mutant cells turn to error-prone, nonhomologous end joining, resulting in genome fragmentation and cell death. Numerous PARP inhibitors show substantial clinical efficacy, including olaparib, niraparib, rucaparib, talazoparib, and veliparib (97–101). Interestingly, clinical benefit from PARP inhibition appears to be derived from PARP “trapping” on DNA rather than inhibition of catalytic activity (102, 103). Additionally, certain patients without *BRCA1/2* mutations respond to PARP inhibition, including at least some patients with mutations in other HR-related genes (e.g., *RAD51*, *PALB2*) (104–106). Further biomarkers that predict response are necessary; in this regard, HR signature panels, which identify aberrations resulting from genomic instability, have shown promise (107, 108). Finally, although PARP inhibitors are clinically effective, resistance invariably occurs through multiple mechanisms, including mutations that decrease PARP trapping, restoration of HR pathway activity, and replication fork protection preventing fork collapse (109–112).

The tumor suppressor *TP53* is the most commonly mutated gene in cancer. Around 90% of *TP53* mutations are missense mutations occurring preferentially in the DNA binding domain of the protein and result in loss of function and/or gain of function depending on the specific mutation and context (113). The majority of *TP53* missense mutations alter the conformation of the p53 protein and result in its unfolding. Thus, efforts have been made to identify small molecules that can stabilize the native p53 conformation and thus restore p53 functionality (114, 115). APR-246, which has made it furthest in clinical trials, is a prodrug that is converted to electrophilic decomposition products that form covalent bonds with two cysteine residues in p53, resulting in thermostabilization of the mutant protein that favors the WT p53 conformation (116). In preclinical models and early clinical trials, APR-246 was able to restore proper p53 DNA binding and activation of apoptosis (117–119). However, although initially showing promising clinical efficacy, APR-246 failed to meet its primary endpoint in a recent phase III trial. This may have been due at least partially to patient selection, as this trial included patients with any of the multitude of *TP53* mutations rather than a more selected subset of patients with mutations for which a benefit may have been more likely. Other strategies are also being explored to target *TP53* mutations, including using oncolytic viruses able to replicate only in cells without p53 activity, reversing p53 gain-of-function signaling, exploiting synthetic dependencies, and developing vaccines for *TP53* mutant–specific neoantigens (120). Unfortunately, no clinically approved drug targeting *TP53* mutations yet exists.

Targeting tumor suppressors remains challenging. Although synthetic dependencies conferred by mutations in tumor suppressors have been identified and targeted, this strategy has proved difficult for the majority of tumor suppressors. Further research will be necessary to fully elucidate possible targetable synthetic dependencies and to develop alternative approaches for targeting mutations in tumor suppressors.

### Chromatin modifiers.

Targeting mutations in chromatin modifiers has in recent years emerged as a viable therapeutic strategy. Mutations in chromatin modifiers themselves are common in several cancers, and other signatures of epigenetic dysregulation, such as changes in DNA methylation and histone modifications, are pervasive across a variety of cancers. To date, the vast majority of FDA-approved small molecules targeting chromatin modifiers are for indications not based on a genetically defined patient population. These agents, which include inhibitors of DNA methyltransferases and histone deacetylases, have proved effective in certain cancers and are reviewed elsewhere (121). Here, we focus on an emerging class of targeted therapies that are designed to directly target mutations in chromatin modifiers. 

The first FDA-approved drugs indicated for mutations that as a direct consequence cause chromatin alterations were for mutations in the core metabolic enzymes *IDH1* and *IDH2*. The IDH1/2 isoforms are responsible for catalyzing the reversible oxidative decarboxylation of isocitrate to α-ketoglutarate (αKG), producing NADPH as a by-product. Mutations in *IDH1/2* occur frequently in acute myeloid leukemia (AML) and gliomas and are neomorphic mutations that lead to the aberrant production of 2-hydroxyglutarate (2-HG) while consuming NADPH (122). Since 2-HG shares structural similarities to αKG, 2-HG is able to inhibit up to about 70 αKG-dependent enzymes, including the TET family of dioxygenases responsible for DNA demethylation (123). *IDH1/2* mutations are mutually exclusive with *TET* mutations, and both are associated with DNA hypermethylation, indicating possible convergence upon a common oncogenic pathway (124, 125). *IDH1/2* mutations also likely simultaneously act through other oncogenic mechanisms including through inhibition of other αKG-dependent chromatin modifiers, reprogramming of cellular metabolism, and accumulation of ROS (126). Inhibitors of both IDH1 and IDH2 have been developed. The IDH1 inhibitor ivosidenib competes with the cofactor Mg^++^ to prevent active site formation, and the IDH2 inhibitor enasidenib binds and stabilizes the inactive form of the enzyme to prevent catalysis (127, 128). Both inhibitors are able to reduce 2-HG concentrations in the plasma by more than 90%, leading to decreased methylation, terminal differentiation, and cell death of *IDH*-mutant cancer cells (129). Despite considerable activity in AML, the clinical setting in which they are most commonly used, long-term responses are rare (130, 131). Resistance invariably occurs involving IDH isoform switching or mutations in the IDH dimer interface, preventing drug binding (132, 133). In gliomas, frequent *IDH1/2* mutations are also observed but progress is even more limited, possibly owing to insufficient CNS penetration, an irreversible epigenetic landscape, or lack of tumor addiction to *IDH1/2* mutations (134). To overcome some of these challenges, development of CNS-penetrant dual IDH inhibitors, including vorasidenib, which is currently in phase III trials, is an important objective (135).

Mutations in the histone methyltransferase *EZH2* have also been successfully targeted. EZH2 is the catalytic subunit of polycomb repressive complex 2 (PRC2), which is responsible for trimethylation of H3K27, a mediator of transcriptional silencing. Gain-of-function mutations in *EZH2* are common in B cell lymphomas, and loss-of-function mutations are frequent in other hematologic malignancies, indicating context-dependent roles for EZH2 (136). In B cell lymphomas, EZH2 is highly expressed and is required to maintain germinal center formation and prevent plasma cell differentiation (137). *EZH2* gain-of-function mutations cause increased H3K27me3 at target genes, which leads to repression of cell cycle checkpoint genes and genes required for plasma cell differentiation, resulting in a transcriptional profile favoring proliferation and self-renewal (138). The EZH2 inhibitor tazemetostat inhibits EZH2 through competition with its cofactor *S*-adenosyl-l-methionine and is able to restore proper differentiation and induce apoptosis (139, 140). Interestingly, while tazemetostat shows the highest clinical efficacy in *EZH2*-mutant disease, it also provides some benefit in a subset of *EZH2*-WT lymphomas (141). It is likely that these *EZH2*-WT tumors harbor other genetic or epigenetic alterations that sensitize to EZH2 inhibition and/or that EZH2 dependency is “inherited” by lymphoma cells derived from the germinal center, as has been suggested (142).

Targeted therapies against specific mutations in chromatin modifiers are beginning to see clinical success, and it is likely that this class of agents will expand over the next few years. A range of mutations in chromatin modifiers exist in cancer, particularly in hematologic malignancies, and many of these may have potential as drug targets. While mutations in chromatin modifiers are believed to be closely linked to tumor onset and progression, their exact roles in these processes will require future research to dissect. For example, while *IDH1/2* mutations are common early alterations in gliomas, some research suggests that these mutations switch from driver to passenger mutations as disease progresses, potentially explaining observed resistance to IDH inhibitors (134). Likewise in AML, mutations in chromatin modifiers, including in *DNMT3A*, *TET2*, and *ASXL1*, are frequent early mutations, but their roles in disease maintenance, and thus whether they would represent therapeutic targets, remain unclear (143). Moreover, the difficulty in assessing the unknown role of targeting early versus late mutations is exemplified by mutations in chromatin modifiers but is not restricted to this class alone and will require future research to address.

## Concluding remarks

Mutation-specific targeted therapies have demonstrated enormous success over the past two decades and remain an exciting area of research. The next decade will likely see an increase in the number of mutated genes in cancer considered druggable. The development of KRAS G12C inhibitors represents an example whereby a mutation previously considered “undruggable” can be targeted with novel chemistry. Developments in targeted therapies for *TP53* mutations, though they have not yet resulted in an FDA-approved drug, represent another area where creative strategies may be employed. Other strategies and chemical approaches are under investigation for a range of other targets. For example, proteolysis-targeting chimeras (PROTACs) allow targeting of a protein via conjugation of the protein’s ligand to an E3 ubiquitin ligase, resulting in proteasome-mediated degradation of the target protein (144, 145). This approach greatly expands the types of proteins that can be targeted as, unlike with traditional small-molecule inhibitors, there is no need to block a specific catalytic or otherwise critical site. Other mutation-specific targeted therapy strategies, including cancer vaccines, are also in early-stage clinical trials (146).

Both the development and the application of targeted therapies have relied predominantly on NGS-based identification of alterations. We expect that the clinical relevance of NGS will become even greater during the next decade as the expanding arsenal of targeted therapies supports the evolving clinical paradigm of treating cancer based on genomic features and agnostic of cell of origin. Indeed, selection of appropriate patient populations in clinical trials is now in some cases guided more by the presence of a mutation than by even the specific cancer type. Several “basket” clinical trials, including the NCI-MATCH trial, have been designed that group patients based on molecular alterations rather than cancer type (147). Moreover, in addition to the diagnostic role of NGS in tumor biopsies, there is considerable interest in the application of NGS in serial sequencing to track response and resistance to therapy, a strategy aided by the development of liquid biopsy techniques including the sequencing of circulating tumor DNA (148). Further, since mutational profiles alone are unable to predict response, additional NGS modalities including transcriptomic analyses and single-cell approaches are under investigation and will likely be needed in certain contexts to guide appropriate patient selection (149, 150). Overall, however, the ability of NGS to detect mutations in individual patients has outpaced the ability to interpret them. First, interpretation of called mutations can be challenging, as it can be difficult to assess the functional significance of each individual mutation (151–153). Some mutations are passenger mutations, and even among genes known to have driver mutations, variants of unknown significance (VUS) remain a challenge (154). To this end, new approaches such as computational modeling of protein structure to predict pathogenicity of VUS and CRISPR/Cas9 saturation mutagenesis scanning to directly assess VUS function have been developed (155, 156). Second, it is possible for multiple potential targetable mutations to be identified within a single tumor, and the choice of therapy in these situations requires clinical judgment. 

Altogether, impressive progress has been made in the identification and targeting of druggable mutations. Targeted therapies have transformed cancer treatment, replacing cytotoxic chemotherapies in multiple treatment lines for a variety of cancers, and are a new option for patients unable to receive standard chemotherapy. As discussed, challenges remain in both the development and the application of targeted therapies. Ultimately, the successful application of targeted therapies will depend on advances in targeting strategies and a more complete understanding of response and resistance.

## Figures and Tables

**Table 1:**
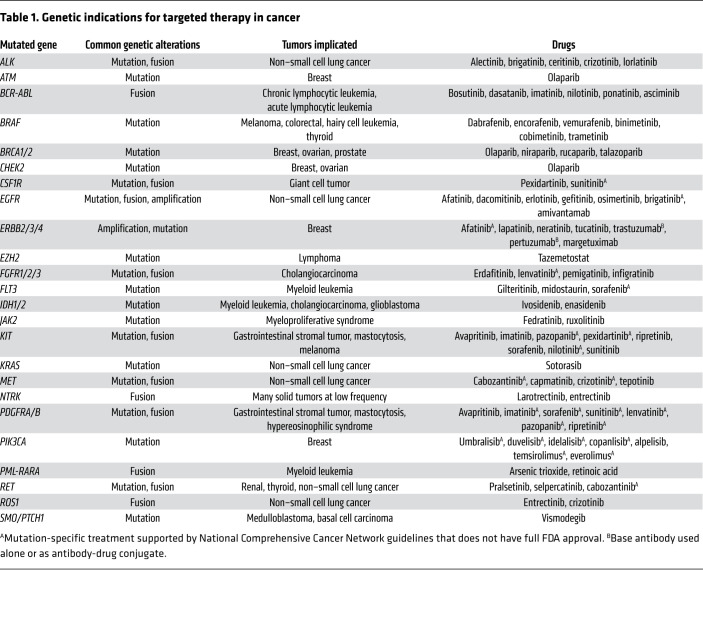
Genetic indications for targeted therapy in cancer

## Version 1 04/15/2022

Electronic publication
